# Hormetic potential of methylglyoxal, a side-product of glycolysis, in switching tumours from growth to death

**DOI:** 10.1038/s41598-017-12119-7

**Published:** 2017-09-15

**Authors:** Marie-Julie Nokin, Florence Durieux, Justine Bellier, Olivier Peulen, Koji Uchida, David A. Spiegel, James R. Cochrane, Craig A. Hutton, Vincent Castronovo, Akeila Bellahcène

**Affiliations:** 1 Metastasis Research Laboratory, GIGA-CANCER, University of Liège, Liège, Belgium; 2 Laboratory of Food Chemistry, Department of Applied Biological Chemistry, Graduate School of Agricultural and Life Sciences, University of Tokyo, Tokyo, Japan; 3 Department of Chemistry, Yale University, 225 Prospect Street, New Haven, Connecticut USA; 4 School of Chemistry and Bio21 Molecular Science and Biotechnology Institute, University of Melbourne, Melbourne, Australia

## Abstract

Metabolic reprogramming toward aerobic glycolysis unavoidably favours methylglyoxal (MG) and advanced glycation end products (AGEs) formation in cancer cells. MG was initially considered a highly cytotoxic molecule with potential anti-cancer value. However, we have recently demonstrated that MG enhanced tumour growth and metastasis. In an attempt to understand this dual role, we explored MG-mediated dicarbonyl stress status in four breast and glioblastoma cancer cell lines in relation with their glycolytic phenotype and MG detoxifying capacity. In glycolytic cancer cells cultured in high glucose, we observed a significant increase of the conversion of MG to D-lactate through the glyoxalase system. Moreover, upon exogenous MG challenge, glycolytic cells showed elevated amounts of intracellular MG and induced *de novo* GLO1 detoxifying enzyme and Nrf2 expression. Thus, supporting the adaptive nature of glycolytic cancer cells to MG dicarbonyl stress when compared to non-glycolytic ones. Finally and consistent with the pro-tumoural role of MG, we showed that low doses of MG induced AGEs formation and tumour growth *in vivo*, both of which can be reversed using a MG scavenger. Our study represents the first demonstration of a hormetic effect of MG defined by a low-dose stimulation and a high-dose inhibition of tumour growth.

## Introduction

Most of the cancer cells favour glucose uptake and process it to lactate to generate their energy. This metabolic switch from oxidative respiration to aerobic glycolysis is commonly known as the Warburg effect^1^. Although described since more than 80 years, it is only in this last decade that intense research efforts attempted to understand how the Warburg effect benefits to cancer cells. It is actually considered that enhanced glycolytic flux in cancer cells notably contributes to rapid ATP production, biosynthesis of building blocks and cell signalling, a set of processes essential for long term uncontrolled cancer cell proliferation and survival^2^. One underestimated consequence of such favoured use of glycolysis by cancer cells is the formation of reactive dicarbonyl species such as methylglyoxal (MG).

MG is a metabolic side-product that is mainly produced following the fifth reaction of the glycolysis through the spontaneous dephosphorylation of glyceraldehyde-3-phosphate (GAP) and dihydroxyacetone phosphate (DHAP)^3,4^. Other cellular sources of MG include sugars, amino acids and acetone (for review ref.^5^). As a highly reactive dicarbonyl molecule, MG interacts with the side chain amino group of arginine and lysine and the thiol group of cysteine residues to form advanced glycation end products (AGEs) such as hydroimidazolones (MG-Hs) and argpyrimidines^6^. MG also glycates lipids and nucleic acids and thus induces major cell dysfunction at proteomic and genomic levels that is referred to as dicarbonyl stress^7^. Glyoxalases 1 and 2 (GLO1 and GLO2) are MG detoxifying enzymes that contribute to control MG level and cytotoxicity by efficiently converting MG to D-lactate in the presence of reduced glutathione (GSH). GLO1 overexpression has been reported in several cancer types among which breast^8^, melanoma^9^ and colon cancers^10^. Other enzymes that have been reported to convert MG into non-toxic compounds include aldo-keto reductase family (AKRs)^11^. Although their activity has been considered negligible compared to the glyoxalase system^12,13^, a recent study has highlighted AKR activity induction as a compensatory mechanism upon GLO1 loss in immortalized murine Schwann cells^14^.

Based on its potent cytotoxic effects, MG has been tested in preclinical settings as a potential therapeutic agent against cancer in the early ‘70 s. MG intra-peritoneal or intra-venous injection to tumour-bearing mice showed a significant reduction of tumour size^15–17^. However, the high toxicity of MG to normal cells excluded any potential development in human therapy. Other strategies to induce high MG stress in cancer cells included the use of cell permeable GLO1 inhibitors such as S-*p*-bromobenzylglutathione cyclopentyl diester (BBGC)^18,19^. Sakamoto and collaborators demonstrated that BBGC selectively induced apoptosis in human lung cancer cells that overexpressed GLO1^20^. Since then, other studies have confirmed the positive correlation between high cellular GLO1 activity and BBGC sensitivity^21^.

Our group is among the first to study dicarbonyl stress in cancer. We have reported the consistent accumulation of MG adducts in breast and colon cancer tumours compared to their normal counterparts^22,23^. More recently, we have demonstrated that GLO1 inhibition in MDA-MB-231 breast cancer cells favours tumour growth and metastases in a xenograft mouse model^24^, thus establishing a functional link between MG stress and tumour progression. Previous to our studies, MG was essentially considered for its pro-apoptotic effects in cancer cells. Accordingly, we have recently proposed the view of a dual role for MG in cancer^25^.

We designed this study in order to explore further the effect of MG stress on cancer cells *in vitro* and *in vivo*. Our data demonstrate that cancer cells responded to both endogenous and exogenous MG stimulus by increasing their D-lactate secretion, an indicator of GLO1 detoxification. We report for the first time that glycolytic cancer cells present a specific response to MG challenge notably consisting of *de novo* expression of GLO1 and Nrf2 at both mRNA and protein levels. Finally, using an *in vivo* tumour model we demonstrated the tumour pro-growth effect of MG at low concentration and its pro-apoptotic effect at high concentration.

## Results and Discussion

### Energetic metabolism characterization of cancer cell lines in relation with endogenous MG level and detoxification potential

**Figure 1:**
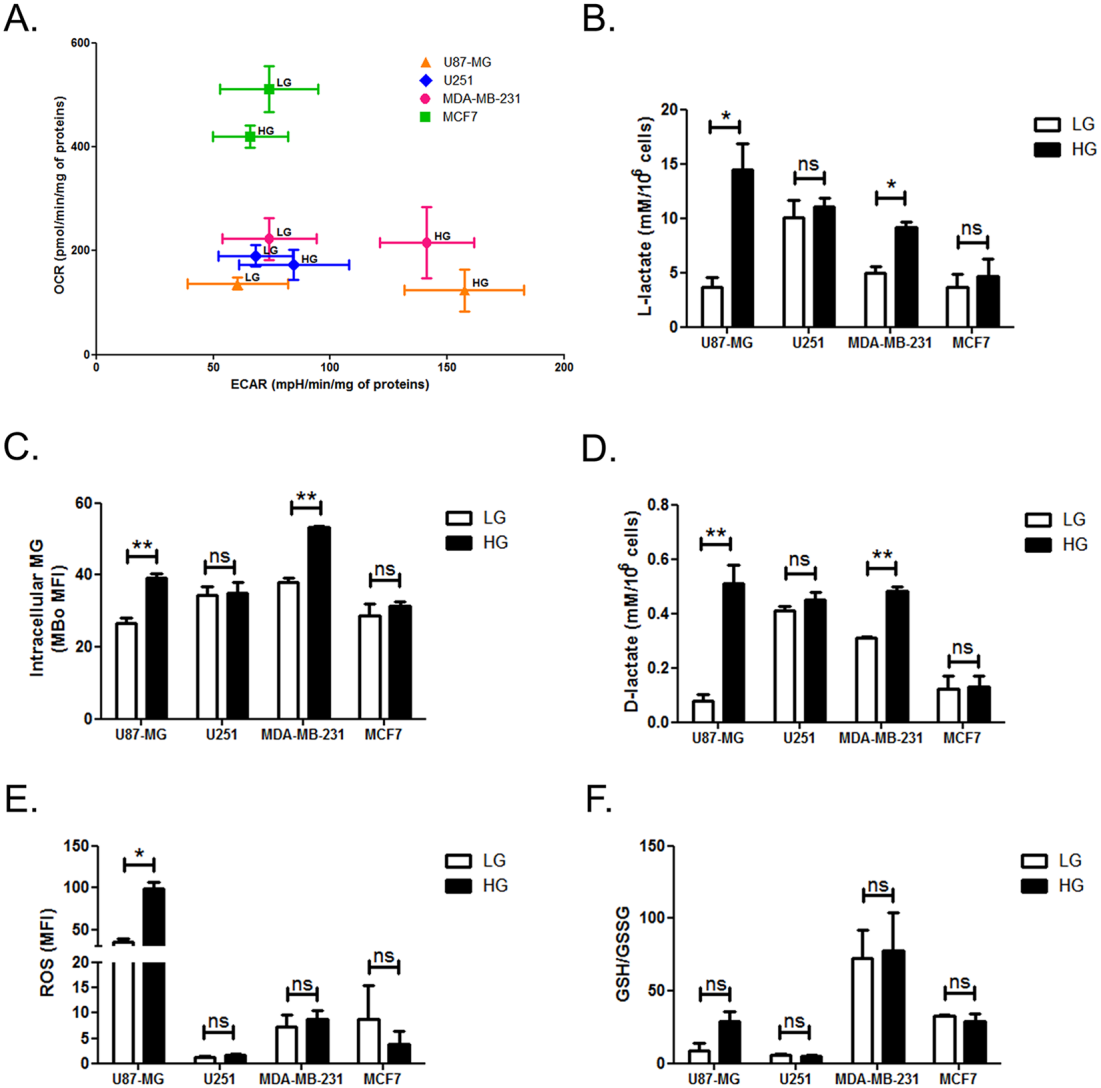
Energetic metabolism characterization and dicarbonyl stress status in cancer cells. U87-MG, U251, MDA-MB-231 and MCF7 cancer cells were cultured in low (LG) or high-glucose (HG) medium. **(A)** Metabolic profiling of the indicated cancer cell lines using Seahorse analyzer showing ECAR (extracellular acidification rate) and OCR (oxygen consumption rate). **(B)** L-Lactate production in 48 h conditioned-medium. **(C)** Intracellular MG was assessed by flow cytometry using MBo specific probe. **(D)** D-Lactate production in 48 h conditioned-medium. **(E)** Reactive oxygen species (ROS) accumulation was assessed by flow cytometry using DCFDA probe. **(F)** GSH and GSSG concentrations were assessed in cell pellets and GSH/GSSG ratio are shown. Data are presented as mean values ± SEM of three independent experiments. *p < 0.05, **p < 0.01 and ns = not significant.

The Warburg effect through which most cancer cells preferentially use glycolysis is expected to have a major impact on the amount of intracellular MG. To our knowledge, the potential relationship between the two has not been thoroughly explored. We first undertook the characterization of the energetic metabolism in GBM (U87-MG and U251) and breast cancer (MDA-MB-231 and MCF7) cell lines cultured under low (LG) and high glucose (HG) conditions. The metabolic profile diagram generated using the Seahorse extracellular flux analyser (Fig. 1A) recapitulates oxygen consumption rates (OCR) and extracellular acidification rates (ECAR) of the cell lines under study. MCF7 cells consistently showed a higher mitochondrial respiration than MDA-MB-231 that is typically considered a glycolytic cancer cell line. No major differences were observed in term of OCR between LG and HG cultured cancer cells. Upon LG culture condition, all the cell lines showed similar glycolytic rates as reflected by ECAR measure. However, when cultured in HG medium only MDA-MB-231 and U87-MG cells increased their glycolytic potential as indicated by the right shift on ECAR axis (Fig. 1A). L-lactate accumulation in the conditioned medium, further confirmed an increased glycolysis flux observed in U87-MG and MDA-MB-231 cells cultured in HG condition (Fig. 1B). Furthermore, L-lactate increase was associated with enhanced intracellular MG levels as detected by FACS using MBo probe, a MG-specific fluorescent sensor in living cells^26^, in both glycolytic cell lines (Fig. 1C).

The measure of D-lactate accumulation in cancer cell conditioned media, as a readout of cellular MG detoxification capacity by GLO1, demonstrated a significant increase of D-lactate production uniquely in U87-MG and MDA-MB-231 glycolytic cells cultured in HG (Fig. 1D). These results are in accordance with the increased glycolytic flux and MG production evidenced in these cells when cultured in HG.

As it has been demonstrated that MG induces oxidative stress in normal and cancer cells^27–31^, we next evaluated redox status in the 4 cancer cells lines cultured in LG and HG. U87-MG cells displayed an elevated basal ROS level when compared to the other cell lines and this level was significantly increased under HG culture condition (Fig. 1E). When considering GSH/GSSG ratio, an indicator of the redox status, MDA-MB-231 had the highest basal content but HG culture did not affect GSH/GSSG ratio, in all the cell lines under study (Fig. 1F).

### MG stress status and GLO1 detoxification capacity of cancer cells in response to MG treatment

**Figure 2:**
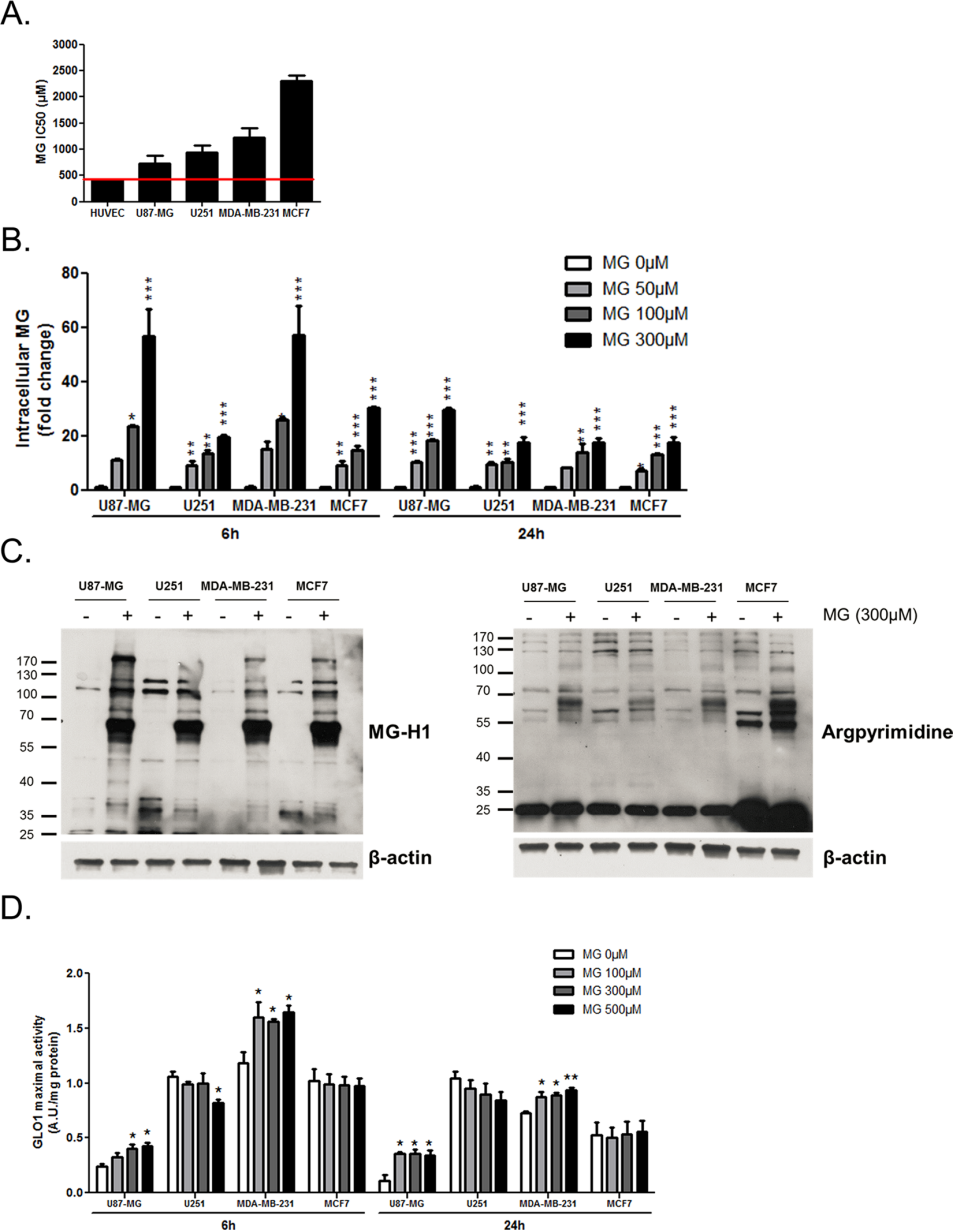
Intracellular MG, MG-adducts levels and GLO1 detoxification capacity in response to MG treatment. U87-MG, U251, MDA-MB-231 and MCF7 cancer cells cultured in low glucose medium were treated with the indicated doses MG **(A)** MG half maximum inhibitory concentration values (IC50) on cancer cell viability. HUVEC normal endothelial cells showed the highest sensitivity to MG compared with cancer cells. **(B)** Intracellular MG production was assessed by flow cytometry using MBo probe in cells treated with the indicated MG concentrations for 6 and 24 h. **(C)** MG-adducts were detected by immunoblotting using specific antibodies against MG-H1 and argpyrimidine residues in cells exposed to MG 300 µM for 6 h, with β-actin as a loading control. Immunoblots are representative of three independent experiments. **(D)** GLO1 maximal activity was measured in cells treated with the indicated MG concentrations for 6 and 24 h, expressed as arbitrary units (A.U.) per mg of proteins. Data are shown as mean values ± SEM three independent experiments. *p < 0.05, **p < 0.01, ***p < 0.001 and ns = not significant, compared with control. Full-length blots are presented in Supplementary Figure 4.

In order to better characterize the response of cancer cells to MG stress, we have next challenged them with increasing doses of exogenous MG. For this purpose, we first determined the half maximal inhibitory concentration values (IC50) of MG on cancer cell viability. We used human umbilical vein endothelial cells (HUVEC) as a normal cell reference which sensitivity to MG has been previously reported^32,33^. Interestingly, IC50 values revealed to be higher in cancer cells when compared to HUVEC (Fig. 2A), pointing to a superior resistance of cancer cells to MG cytotoxicity. MG IC50 values ranged below 1000 µM for GBM cells and were slightly higher for breast cancer cells with MCF7 cells showing the highest IC50 (Fig. 2A). Based on the results obtained under HG culture condition, we would have expected that glycolytic cancer cells might present with the more resistant phenotype toward exogenous MG challenge. One possible explanation for this apparent difference could be that these cells are, in fact, in the presence of higher amounts of free MG than non-glycolytic cells.

To test this hypothesis, we assessed MG intracellular concentrations using MBo specific probe (Fig. 2B). Intracellular MG was significantly elevated in all cancer cells 6 h and 24 h after treatment with increasing doses of MG. Consistent with this effect was the detection of increased amounts of argpyrimidine and MG-H1 specific MG-adducts using western blot in all cancer cells after 6 h of MG treatment (Fig. 2C). Under both basal and MG treatment conditions, MCF7 cells apparently accumulated more argpyrimidine adducts than the other cell lines. We also observed that MCF7 cancer cells presented the highest oxidative metabolism (Fig. 1A), which is generally associated with a low proliferation rate^34^ and a reduced protein turn-over (synthesis and degradation). Moreover, it is known that glycated proteins gain in stability as a result of structural modifications and/or of proteasomal defects, both occurring under MG stress. It is thus likely that MCF7 cells present with a higher amount of long-lived proteins, which might be particularly prone to accumulation of glycation damage. These hypotheses should be further explored using other oxidative cancer cell lines with low proliferation capability.

While all cancer cells dose-dependently increased their intracellular MG level, it is noteworthy that U87-MG and MDA-MB-231 glycolytic cells showed a major MG increase of up to 60-fold (Fig. 2B). MCF7 and U251 cells showed a less marked increase, that reached 20 and 30-fold over their respective basal levels (Fig. 2B). These data establish that glycolytic cells accumulate more intracellular MG when they are in the presence of exogenous MG than non-glycolytic cells. This observation could explain the low MG IC50 values determined for glycolytic cells. Actually, MG IC50 estimation in those cells probably does not accurately reflect their tolerance to an exogenous supply of MG. Altogether, these results let us propose that glycolytic cancer cells have up taken more exogenous MG and/or have produced more MG upon MG treatment. Because MG freely equilibrates across the cytoplasmic membrane^35^, high intracellular MG levels in glycolytic cells more likely result from an effect of MG on glycolytic flux. MG has been previously shown to inhibit GAPDH activity *in vitro* ^36^ thus potentially favouring the accumulation of triose phosphate intermediates and the subsequent spontaneous formation of MG. Interestingly, Beisswenger and collaborators reported an inverse relationship between GAPDH activity and MG production in type 1 and 2 diabetic patients^37^. Considering that the expression/activity status of GAPDH can be deregulated in cancer cells^38^, it would be interesting to evaluate MG stress in this context.

After 24 h of treatment, intracellular amounts of MG were equilibrated in all cell lines to 17 to 30-fold over their basal levels (Fig. 2B). We reasoned that glycolytic cells might present with a more efficient detoxification capacity than non-glycolytic cancer cells. To evaluate this possibility, we next measured GLO1 maximal activity in these cells. We found that MDA-MB-231 and U87-MG cells significantly adapted their GLO1 detoxification capacity in the first 6 hours after exposure to MG when compared with MCF7 and U251 cells. For the latter cell lines, GLO1 maximal activity stayed constant and even decreased in the presence of the highest concentration of MG tested (Fig. 2D).

### Glycolytic cancer cells express increased amounts of GLO1 and Nrf2 in the presence of exogenous MG

**Figure 3:**
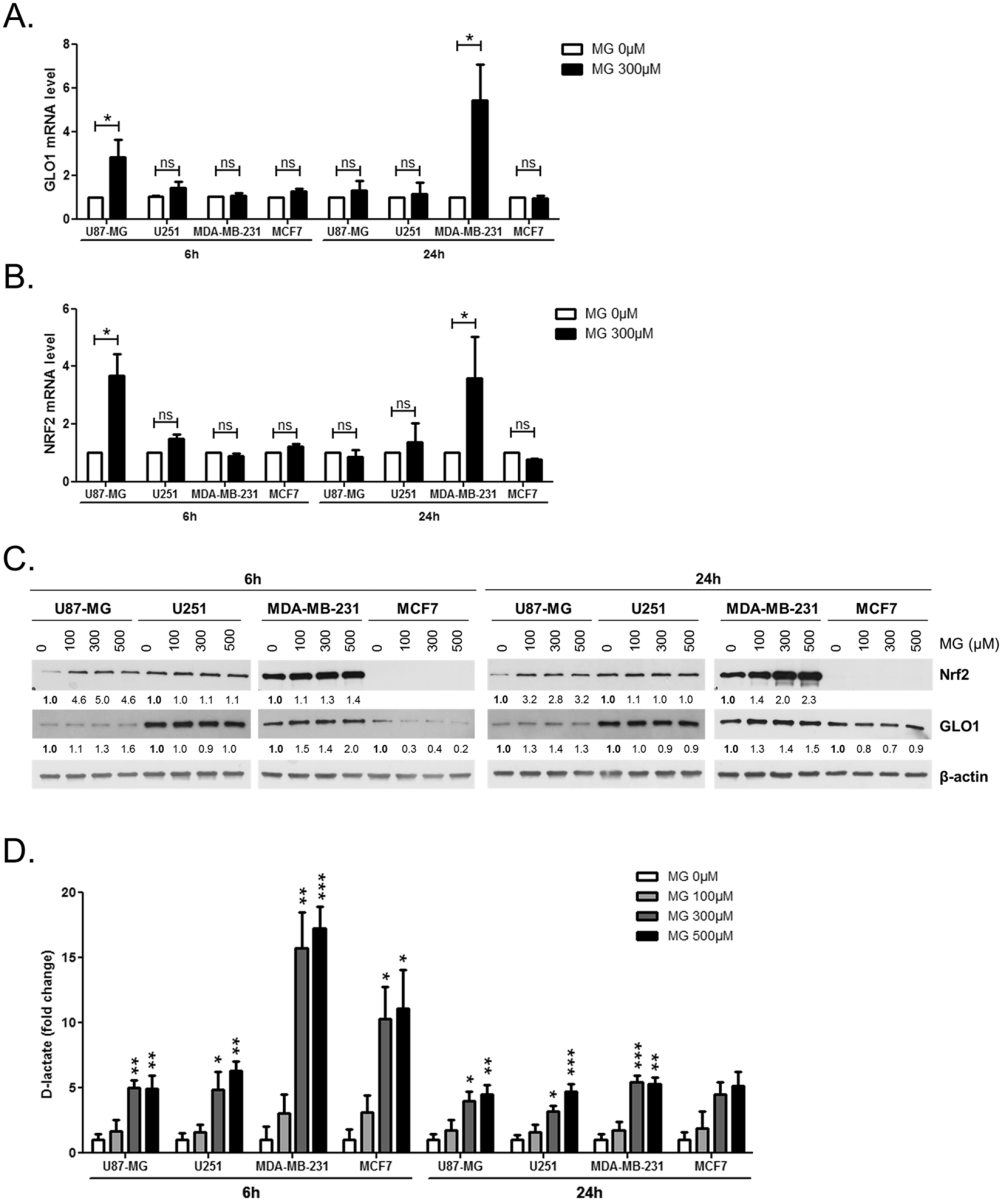
Glycolytic cancer cells expressed increased amounts of GLO1 and Nrf2 upon MG treatment. U87-MG, U251, MDA-MB-231 and MCF7 cells cultured in low glucose medium were treated with the indicated doses of MG. **(A)** GLO1 and **(B)** NRF2 mRNA levels were assessed in response to MG treatment by RT-qPCR. **(C)** GLO1 and Nrf2 protein levels were quantified using immunoblotting, with β-actin as a loading control. Numbers represent fold increase relative to the control condition shown in bold. Immunoblots are representative of three independent experiments. **(D)** D-lactate production in conditioned-medium was assessed after 6 and 24 h MG treatment. Data are presented as mean values ± SEM of three independent experiments. *p < 0.05, **p < 0.01, ***p < 0.001 and ns = not significant, compared with control. Full-length blots are presented in Supplementary Figure 5.

Data gathered so far indicate that exogenous MG rapidly induces the accumulation of detectable protein adducts and support the hypothesis according to which glycolytic cancer cells present with a more efficient MG detoxification capability than non-glycolytic ones. Validating further this hypothesis, we found that increased GLO1 activity was accompanied by *de novo* expression of GLO1 mRNA (Fig. 3A) and protein (Fig. 3C) only in U87-MG and MDA-MB-231 cells. Thus, supporting the adaptive nature of glycolytic cancer cells to high MG stress when compared to non-glycolytic ones. Nrf2 stress-responsive transcription factor has been shown to exert a positive transcriptional control on *GLO1* gene expression^39^. We next showed that Nrf2 expression was also increased at both the mRNA (Fig. 3B) and protein (Fig. 3C) levels upon MG treatment in U87-MG and MDA-MB-231 cells. This observation is compatible with an Nrf2-induced *de novo* regulation of GLO1 expression in glycolytic cancer cells upon MG stress. MG has been reported to induce reactive oxygen species (ROS) formation in cancer cells^30^ and ROS can trigger Nrf2 expression^40^. Therefore, we evaluated ROS levels in parallel treatment experiments. MG treatment conditions did not influence ROS basal levels in all cells under study as measured using oxidized DCFDA detection by flow cytometry (Supplementary Figure 1).

We have observed an enhanced ability of glycolytic cancer cells to convert MG into D-lactate when cultured in HG medium (Fig. 1D). Next, we asked whether increased GLO1 detoxification capacity would be associated with augmented D-lactate production upon MG treatment. Six hours after MG challenge, all cell lines showed an increased D-lactate production with the highest one (15-fold above basal level) observed in MDA-MB-231 cells (Fig. 3D). After 24 h, D-lactate production was comparable in all cell lines and maintained at maximum 5-fold above the corresponding basal levels (Fig. 3D). Altogether these data indicate that increased MG production, associated with elevated glycolytic flux, is efficiently detoxified by GLO1 into D-lactate in cancer cells. Glycolytic cancer cells showed an enhanced GLO1 detoxifying capacity notably through the induction of GLO1 and Nrf2 expression. It is remarkable that glycolytic cells in the presence of endogenous MG (HG condition) are challenged with at least 10-fold less MG than under exogenous treatment. Increased MG detoxification to D-Lactate occurred in both cases but was not accompanied by an increase of GLO1 activity/expression under low MG (Supplementary Figure 2). Thus suggesting for the first time that glycolytic cancer cells might be able to sense MG stress level and to adapt their detoxification capacity accordingly.

### Other MG detoxifying enzymes are expressed in cancer cells and compensate for the loss of GLO1 activity

**Figure 4:**
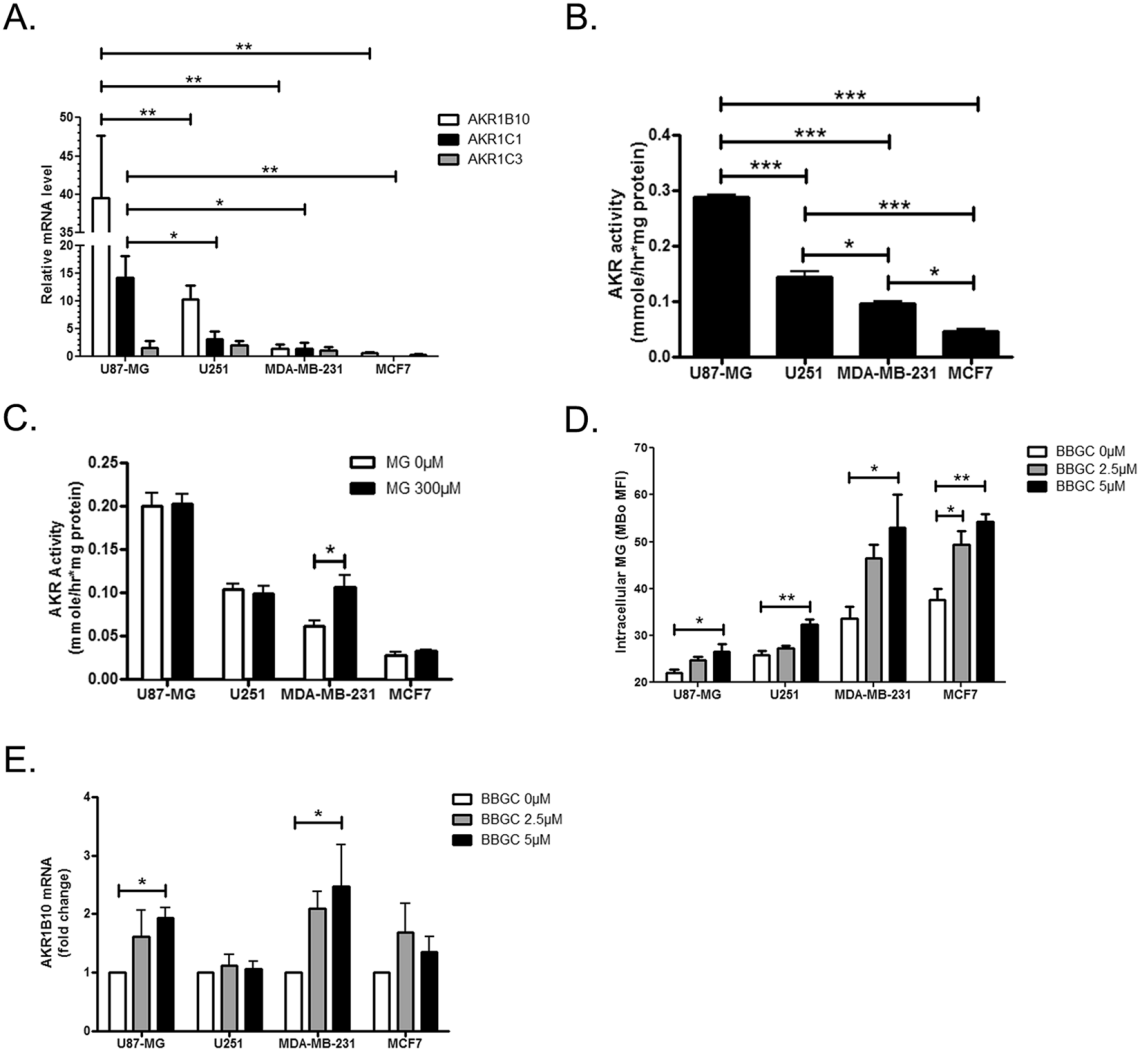
Aldo-keto reductases (AKRs) detoxifying enzymes are expressed in cancer cells and compensate for the loss of GLO1 activity. (**A**) U87-MG, U251, MDA-MB-231 and MCF7 cells were cultured in high-glucose medium and their mRNA levels for AKR1B10, AKR1C1 and AKR1C3 were evaluated by RT-qPCR. mRNA levels are shown as relative to MDA-MB-231 cells. (**B**) Basal AKR activity is shown as mmole of NADPH converted per h per mg of protein in the indicated cancer cells. (**C**) AKR activity was measured in the indicated cell lines treated with MG 300 µM during 24 h. (**D**) Intracellular MG was assessed by flow cytometry using MBo probe after 48 h treatment with BBGC at the indicated doses. (**E**) AKR1B10 mRNA levels were assessed by RT-qPCR in BBGC treated cells. Data are presented as fold change relative to untreated cells. All data are shown as mean values ± SEM of three independent experiments. *p < 0.05, **p < 0.01, ***p < 0.001.

Although both U87-MG and MDA-MB-231 cells responded to high MG stress by increasing their GLO1 expression and activity, we noticed that U87-MG cells did not produce as much D-lactate as MDA-MB-231 cells. As a cofactor of GLO1 enzyme, GSH cellular content might be limiting for GLO1 activity rate upon MG stress. U87-MG cells have a significantly lower GSH/GSSG basal level when compared with MDA-MB-231 cells (Fig. 1F). Altogether, these observations let us envisage the possibility that U87-MG cells may use other MG detoxification enzymes such as aldo-keto reductases (AKRs). AKR family members such as AKR1B and AKR1C have been previously associated with cancer cell proliferation and resistance to chemotherapy in GBM and breast cancer^41,42^. In order to explore this hypothesis, we first assessed AKR gene expression in the cancer cell lines under study. Basal levels of AKR1B10 and AKR1C1 mRNA were higher in GBM cells than in breast cancer cells, with U87-MG cells significantly displaying the highest amounts (Fig. 4A). Global AKR activity assessed in these cells was consistent with their mRNA expression levels (Fig. 4B). In response to MG treatment, AKR activity increased in MDA-MB-231 cells while it remained stable in U87-MG cells which displayed a high basal expression level of AKRs (Fig. 4C).

To further explore the possibility of a compensatory mechanism based on AKR enzymes expression in cancer cells, we induced GLO1 activity deficit in these cells using increasing concentrations of BBGC inhibitor. Whereas basal intracellular MG levels were lower in GBM than in breast cancer cells, they increased significantly in all cell lines in the presence of 5 µM BBGC (Fig. 4D). Finally, AKR1B10 mRNA levels showed a significant increase in both U87-MG and MDA-MB-231 cells upon BBGC treatment thus suggesting an adaptation to GLO1 activity loss in these cells (Fig. 4E).

We have demonstrated that glycolytic cancer cells are more prone to respond to MG challenge through increased Nrf2 and MG-detoxifying enzymes expression. Therefore, we next envisaged that low doses of MG might be beneficial to tumour growth while high concentrations would be cytotoxic.

### Cancer cells display a characteristic biphasic dose response growth curve upon MG treatment

**Figure 5:**
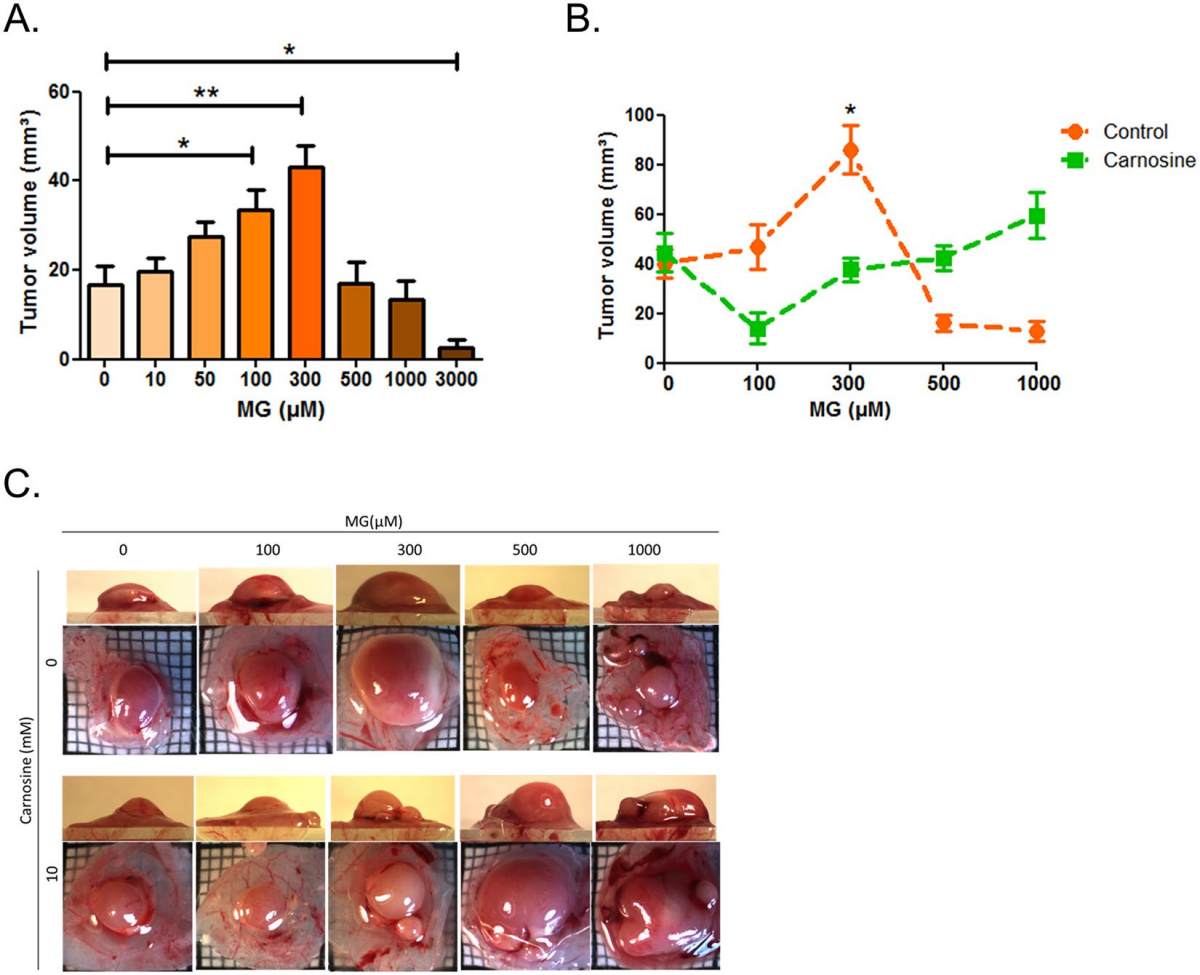
Cancer cells display a biphasic dose response growth curve upon MG treatment. U87-MG cells were grown on the chicken chorioallantoic membrane (CAM) and treated daily with **(A)** the indicated doses of MG and/or **(B)** carnosine 10 mM. After 7 days, tumour volumes were calculated. **(C)** Top and profile views of representative experimental CAM tumours. At least 10 eggs were used for each experimental condition. Data are mean values ± SEM. *p < 0.05 and **p < 0.01.

In order to test the possibility that MG could exert a dual effect on tumour growth, we have challenged U87-MG human glioblastoma cancer cells engrafted on the chicken chorioallantoic membrane (CAM) with increasing concentrations of MG. We observed an increase of tumour volume in the presence of low doses of MG that reached significance for 100 and 300 µM when compared to untreated tumours (Fig. 5A), as shown for representative tumours in Fig. 5C. At higher doses (from 500 to 3000 µM), tumour volume showed a significant decrease (Fig. 5A and C). The biphasic profile of the graph shown in Fig. 5B highlighted a hormetic dose effect of MG on tumour growth. Similar results were obtained with MDA-MB-231 breast cancer cells using the same *in vivo* CAM model (Supplementary Figure 3).

**Figure 6:**
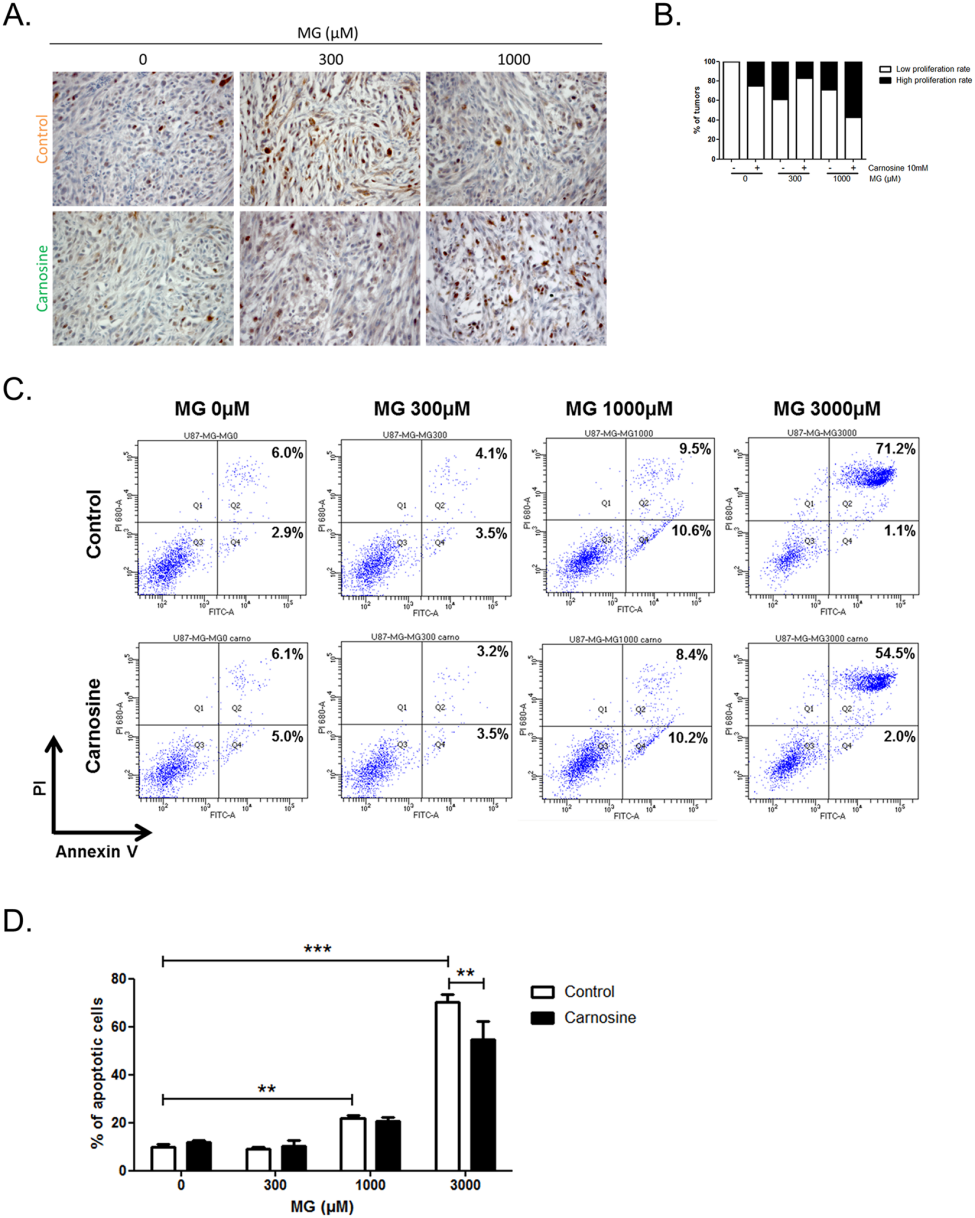
Proliferative and apoptotic effects of MG and carnosine treatment on CAM tumours. Experimental tumours shown in Fig. 5 were subjected to immunohistochemical staining of Ki67 proliferation marker. A representative picture of Ki67 staining is shown in **(A),** and the proportion of tumours discriminated in low and high proliferation rate among the different conditions is represented in panel **(B).** Apoptosis analysis in cells treated with MG at the indicated doses with or without co-treatment with carnosine 10 mM. Representative flow cytometry dot-plots are shown in **(C)** and annexin V positive cells are quantified in **(D).** Data are presented as mean values ± SEM of three independent experiments. **p < 0.01, ***p < 0.001.

**Figure 7:**
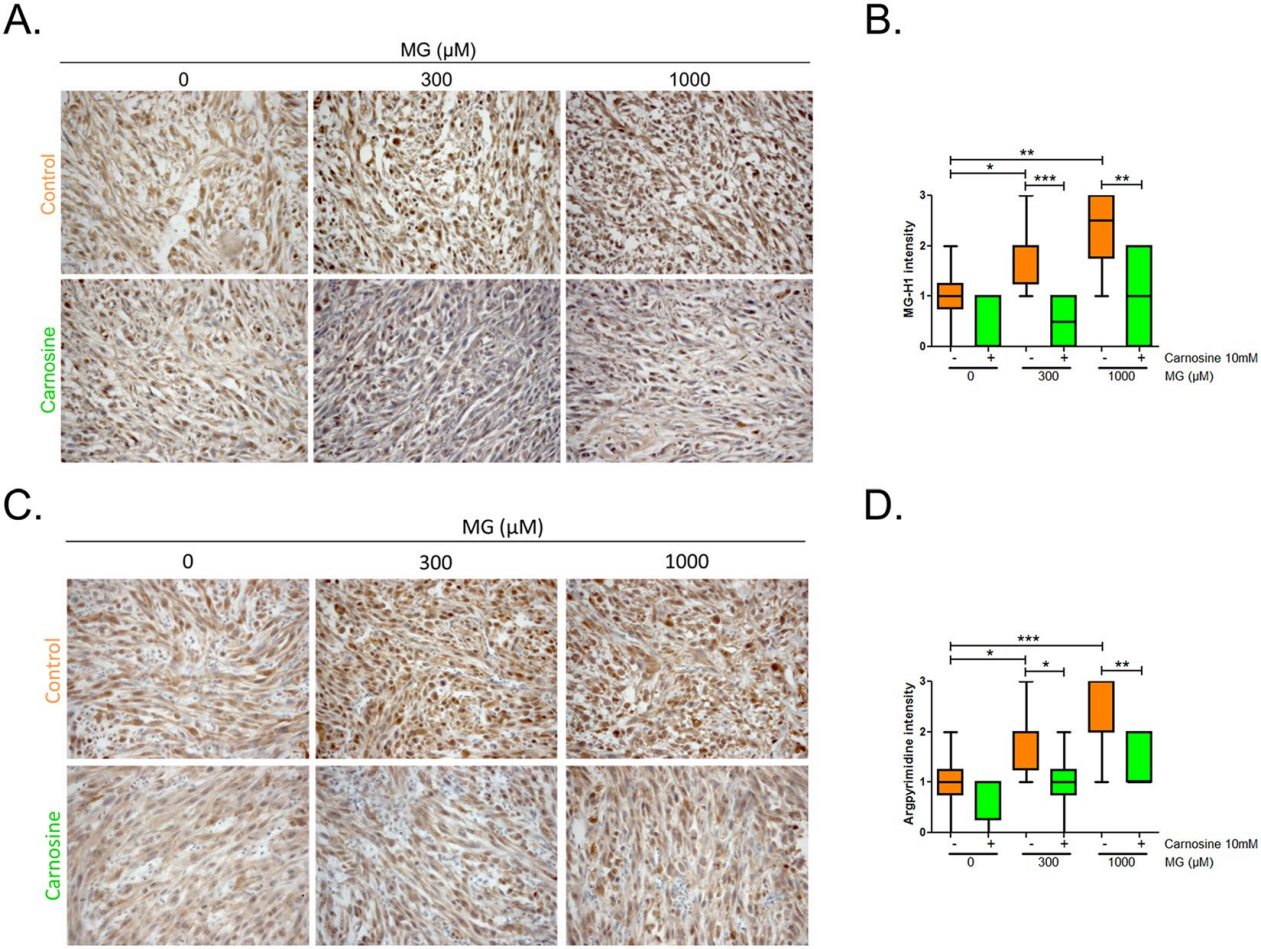
Accumulation of MG-adducts in CAM tumours. Experimental tumours shown in Fig. 5 were subjected to immunohistochemical staining of MG-H1 and argpyrimidine MG-adducts. Representative pictures **(A**,**C)** and immunostaining quantification **(B**,**D)** of MG-H1 and argpyrimidine staining are shown, respectively. *p < 0.05, **p < 0.01, ***p < 0.001.

We next reasoned that both pro and anti-growth MG effects on tumours should be reversed using MG scavenger molecules. Using the same *in vivo* tumour growth model, the co-treatment with MG and carnosine, a potent natural MG scavenger, significantly reversed cancer cell response to MG (2-way anova, p < 0.001) (Fig. 5B), and as shown for representative tumours in Fig. 5C. In good accordance with the observed increase of tumour volume, we found a higher proportion of tumours displaying Ki67 proliferation marker positive cells in CAM tumours treated with 300 µM MG when compared with either untreated tumours or tumours treated with 1000 µM MG (Fig. 6A,B). Apoptotic rates estimated under the same conditions showed that apoptosis is significantly induced at 1000 µM MG in U87-MG cells. MG pro-apoptotic effect is reversed under carnosine treatment at 3000 µM MG (Fig. 6C,D). Using immunohistochemistry, we further confirmed the dose-dependent increase of MG-H1 (Fig. 7A,B) and argpyrimidine (Fig. 7C,D) MG-adducts in the experimental CAM tumours. Carnosine treatment efficiently inhibited MG-H1 and argpyrimidine adducts accumulation (Fig. 7A,B and Fig. 7C,D, respectively).

Altogether, these data demonstrate for the first time that, in a specific hormetic window, MG favours tumour growth *in vivo*. It is noteworthy, that carnosine treatment potently reversed both pro- and anti-growth effects exerted by MG on malignant tumours *in vivo*. We believe that these observations bear a significant interest for the development of future preventive or therapeutic anti-cancer strategies.

## Concluding Remarks

Hormesis is defined as “a process in which exposure to a low dose of a chemical agent or environmental factor, that is damaging at higher doses, induces an adaptive beneficial effect on the cell or organism”^43^. We demonstrate for the first time the hormetic effect of MG on cancer cell growth and reconcile seemingly contrasting earlier data accumulated on MG. In a non-tumoural context, a dual role has been previously demonstrated for MG that was favourable to neurons viability and excitability at low levels while high levels were cytotoxic^44^. Importantly, our data demonstrate that cancer cells are not equal when facing MG stress and this has probably contributed to the existing controversy around MG role in cancer. Glycolytic cancer cells cultured under high glucose condition produce MG and show an increased detoxification capacity when compared with cells cultured in low glucose medium. More remarkable is the induction of Nrf2 and GLO1 expression in the presence of high MG stress in glycolytic cells leading to efficient MG conversion to D-Lactate. In this study, we demonstrate for the first time that glycolytic cancer cells are able to induce Nrf2 and GLO1 expression upon MG stress. Menegon and collaborators^45^ have recently reviewed the dual roles of Nrf2 in cancer. The main function of this transcription factor is to activate the antioxidant cellular response to protect cells from oxidative stress. However, Nrf2 not only protects normal cells from stress but also cancer cells, supporting the idea that it could be an oncogene. It is remarkable that GLO1 also plays a dual role in cancer. On the one hand, others^46^ and we^24^ have shown that GLO1 acts as a tumour suppressor and its loss has been linked with tumour growth and metastasis development *in vivo*. On the other hand, GLO1 is overexpressed and/or amplified in tumours and its loss has been associated with MG-induced cytotoxicity and apoptosis^20,47–52^. In fact, Nrf2 and GLO1 favour the survival of cancer cells by protecting them from excessive dicarbonyl and/or oxidative stress, both of which have been implicated in cancer initiation and progression.

We demonstrated that the adaptive behaviour of cancer cells to MG stress favours growth and survival that can be efficiently reversed by carnosine. This naturally occurring dipeptide has been shown to exert anti-cancer effects^53,54^ and ongoing studies in our laboratory examine its effects on glioblastoma and colon cancer tumour growth in mouse models. Next to carnosine, our results bring new interest in other MG scavengers such as metformin and aminoguanidine as anti-cancer agents.

As mentioned above, dicarbonyl stress is linked to oxidative stress by many aspects. One of them is that MG stress induces ROS production in normal and cancer cells. Another key common feature is Nrf2 transcription factor. Nrf2 is often referred to as the main activator of cellular antioxidant response but it is also the main regulator of GLO1 and AKRs expression thus playing a central role in cell response to MG stress^39,55^. We have shown that cancer cells express AKRs that represent a compensatory mechanism in case of GLO1 loss or decreased activity. Future studies addressing the role of MG dicarbonyl stress in cancer cells will have to assess energy metabolism and oxidative status in parallel with MG stress cellular adducts and detoxification capacity.

Metabolic reprograming is an important hallmark of proliferating cancer cells. The preferential use of glycolysis unavoidably generates MG, which level must be strictly adjusted to be kept in a subtoxic range in glycolytic cancer cells. In this study, we demonstrate that maintaining tolerable MG stress turns out to be beneficial to cancer cells through both resistance to apoptosis and enhanced growth.

## Materials and Methods

### Cell culture and reagents

U87-MG, U251, MDA-MB-231 and MCF7 cancer cell lines were obtained from ATCC (American Type Culture Collection, Manassas, VA) and grown in DMEM medium (Lonza, Basel, Switzerland) supplemented with 10% fetal bovine serum (FBS; ThermoFischer Scientific, Waltham, MA) and 2 mM L-Glutamine (Lonza). Glucose concentration was either 4.5 g/L (high glucose condition) or 1 g/L (low glucose condition). HUVEC cells were isolated following a method described in Jaffe *et al*.^56^. HUVEC cells are grown in MCDB131 medium (Invitrogen, Carlsbad, CA) supplemented with 20% FBS, 2 mM L-Glutamine, 50 µg/mL heparin (Sigma, St. Louis, MO), ECGS 50 µg/mL (BD Biosciences, Franklin Lakes, NJ) and 50UI/50 µg/mL penicilline/streptomycin (Lonza). For seeding of HUVEC cells, plates are coated with 0,2% gelatin (Sigma). Methylglyoxal (MG, Sigma) treatment was performed during 6 or 24 h in low glucose medium. Methylglyoxal solution contamination by formaldehyde was assessed by NMR analysis and considered insignificant (<3%)^24^. BBGC, S-*p*-bromobenzylglutathione cyclopentyl diester, a cell-permeable GLO1 inhibitor, was synthesized as described^18^. BBGC treatment was performed for 48 h in high glucose medium. Anti-argpyrimidine antibody (mAb6B) specificity has been previously confirmed by competitive ELISA and was shown to not react with other MG-arginine adducts such as 5-hydro-5-methylimidazolone and tetrahydropyrimidine^57^. MBo (Methyl diaminobenzene-BODIPY) is a cell permeable MG specific probe, synthetized and validated as described^26^.

### Seahorse analysis

Oxygen consumption rate (OCR) and extracellular acidification rate (ECAR) of U87-MG, U251, MDA-MB-231 and MCF7 cells cultured in low and high glucose medium were determined using Seahorse XFp bioenergetic analyzer (Agilent) according to manufacturer’s recommendations. Briefly, cells were plated in Seahorse microplates (15000, 5000, 15000 and 20000 cells/well for U87-MG, U251, MDA-MB-231 and MCF7 cells, respectively). Medium was replaced by DMEM without glucose supplemented with 2 mM glutamine 1 h before the measurements. D-glucose was added to a final concentration of 10 mM and OCR and ECAR were measured. Data were normalized to protein quantity.

### L- and D-Lactate dosage

L- and D-Lactate concentrations were assessed in conditioned medium, the number of cells in each condition was counted for normalization. Conditioned medium (diluted 3 times for L-Lactate measure) was incubated in the presence of NAD+, hydrazine and L- or D-Lactate dehydrogenase enzyme (Sigma). Lactate concentration was determined by comparing NADH formation measure at 320 nm to the absorbance of a calibration curve. L- and D-Lactate concentrations were normalized per million cells.

### Cellular MG quantification

MBo (Methyl diaminobenzene-BODIPY) was used to detect endogenous MG. The cells were treated with 5 mM MBo in complete medium as described^26^. After 1 h, the cells were washed with PBS and incubated in low- or high-glucose medium for 6 h or treated in low-glucose medium with MG for 6 to 24 h. For MG induction after BBGC treatment, cells were treated 48 h with BBGC, then 1 h with MBo. Then cells were washed and incubated for more 6 h in the presence of BBGC. Cells were then trypsinized and analyzed by flow cytometry (FACSCanto, BD Biosciences). Data are represented as mean ± SEM of at least 3 biological replicates.

### ROS measurement by FACS

ROS production was measured using CM-H2DCFDA fluorescent probe (Invitrogen) according to the manufacturer’s protocol. Briefly, cells were trypsinized and incubated with CM-H2DCFDA probe (diluted 1/5000 in HBSS, Invitrogen) for 15 min in the dark. After centrifugation, cells were incubated in culture medium during 15 min at 37 °C before FACS analysis.

### Glutathione (GSH) and glutathione disulfide (GSSG) levels

GSH/GSSG ratio were determined as described^58^. Briefly, cell pellets were extracted in 0.1% Triton-X (Sigma) and 0.6% sulfosalicylic acid (Sigma) in KPE buffer (0.1 M potassium phosphate buffer with 5 mM EDTA disodium salt, pH 7.5) and sonicated in icy water for 3 min. After 2 freeze-thaw cycles, lysates were centrifuged at 3000 g for 4 min and supernatants were collected. For total GSH measurement, supernatants were mixed with DTNB solution and glutathione reductase to convert GSSG to GSH and then β-NADPH was added. The rate of 2-nitro-5-thiobenzoic acid formation was followed spectrophotometrically at 412 nm for 2 min. Total GSH concentrations were determined based on the values obtained from the standard curve. For GSSG measurement, supernatants were mixed with 2-vinylpyridine to derivatize GSH for 1 h and were then neutralized by adding triethanolamine for 10 min. The derivatized samples were analyzed as described for total GSH dosage. GSH concentrations were calculated using the following formula: [GSH] = [GSH_total_] − 2*[GSSG] and allowed the determination of GSH/GSSG ratio.

### Methylglyoxal inhibitory concentration 50 (IC50) determination

Cells were plated in 24-well plate and treated for 24 h with increasing doses of MG. Cells were then washed, lysed by sonication and their DNA content was assessed using bisbenzimide (Sigma) incorporation. DNA content was detected spectrophotometrically by excitation at 360 nm and fluorescence emission at 460 nm. IC50 was determined as the concentration of MG able to decrease by half the quantity of DNA detected.

### Western Blot

Cell extraction was performed in 1% SDS buffer containing protease and phosphatase inhibitors (Roche, Penzberg, Germany). Protein concentration is determined using bicinchoninic acid assay (Pierce, Carlsbad, CA). Twenty µg of protein were separated on 10 or 12.5% SDS-PAGE and transferred to PVDF membranes (Roche). Blocking was performed in 5% non-fat dried milk (Biorad, Hercules, CA) in TBS-Tween 0.1% for 1 h. Membranes were then incubated overnight at 4 °C with primary antibodies: Argpyrimidine (1/10,000, mAb6B), MG-H1 (1/1000, STA-011, Cell Biolabs), Nrf2 (1/1000, Ab62353, Abcam), GLO1 (1/1000, #02-14, BioMac, Leipzig, Germany), β-actin (1/5000, A5441, Sigma). Membranes were then incubated for 1 h in the presence of the appropriate secondary antibody coupled to horseradish peroxidase. Immunoreactive bands were detected using ECL Western Blotting substrate (Pierce). Bands quantification by densitometry and normalization to ß actin was performed using ImageJ software (NIH, Bethesda, MD).

### RNA isolation and quantitative reverse transcription-PCR (qRT-PCR)

RNA extraction was done according to the manufacturer’s protocol (NucleoSpin RNA, Macherey-Nagel, Düren, Germany). Reverse transcription was performed using the Transcription First Strand cDNA Synthesis Kit (Roche). Hundred ng of cDNA were mixed with primers, probe (Universal ProbeLibrary System, Roche) and 2x Takyon Rox Probe MatserMix dTTP Blue (Eurogentec, Seraing, Belgium) or Fast Start SYBR Green Master Mix (Roche). Q-PCR were performed using the 7300 Real Time PCR System and the corresponding manufacturer’s software (Applied Biosystems, Carlsbad, CA). Relative gene expression was normalized to 18 S rRNA. Primers were synthesized by IDT (Coralville, IA) and their sequences are detailed in Supplementary Table 1.

### GLO1 activity assay

GLO1 activity assessment was performed as we previously described^22,24^. Briefly, S-D-lactoylglutathion formation was followed at 240 nm in a reaction mixture composed of pre-incubated MG with reduced glutathion (Sigma) and protein extracted in RIPA buffer. GLO1 maximal activity measure is expressed as arbitrary units (A.U.) of enzyme per mg of proteins.

### Chicken chorioallantoic membrane (CAM) tumour assay

On embryonic day 11, a suspension of 5 × 10^6^ cells in culture medium alone for U87-MG cells or 2 × 10^6^ cells mixed (1:1) with matrigel (BD Biosciences) for MDA-MB-231 cells was deposited in the center of a plastic ring on the chick chorioallantoic membrane. Treatment with methylglyoxal alone or combined with Carnosine (Sigma) in saline solution was performed daily from the day after cells implantation to the end of the experiment. Tumours were collected at embryonic day 18 and fixed in 4% paraformaldehyde for histology analysis. Tumour size was measured with a caliper and tumour volume was calculated with the formula 4/3π × H/2 × L/2 × W/2, with H, L and W standing for height, length and width, respectively.

### Immunohistochemistry

Formalin-fixed paraffin embedded sections were deparaffinized and rehydrated. Endogenous peroxidase activity was inhibited by a 30 min bath in methanol containing 3% hydrogen peroxide. Antigen retrieval was obtained by a 40 min bath in 10 mM sodium citrate buffer pH6 at 95 °C. Non-specific binding was avoided by incubation with 1,5% normal serum (Vector Laboratories, Burlingame, CA) for 30 min. Then, primary antibody, either mouse anti-Argpyrimidine (1/10.000), mouse anti-Ki67 (1/100, Dako) or rabbit anti-MG-H1 (1/500, STA-011 Cell Biolabs, San Diego, CA) was applied for the night. Sections were next incubated with anti-mouse or anti-rabbit biotinylated secondary antibody (Vector Laboratories) for 30 min followed by staining with 3,3′ diaminobenzine tetrachloride (DAB). After counterstaining with hematoxylin, slides were dehydrated and mounted with DPX (Sigma). Control slides incubated without primary antibody showed no immunoreactivity.

### Immunohistochemical staining evaluation

The immunostaining was assessed and scored by two independent examiners. Argpyrimidine and MG-H1 scores were attributed according to the intensity of the staining (0, 1+, 2+, 3+). Ki67 immunostaining was evaluated as the percentage of positive nucleus present in the sections. Then two categories were distinguished, low proliferative rate for the cases presenting ≤30% of positive nucleus and high proliferative rate for the cases presenting >30% of positive nucleus.

### Apoptosis

Apoptosis was measured with the FITC-Annexin V apoptosis Detection Kit I (BD Biosciences) by FACS according to manufacturer’s instructions.

### Aldo-keto reductase activity assay

AKR activity was assayed in PBS 100 mM pH7.2 at 37 °C as previously reported^14^. Protein extraction was performed with ice-cold cytosolic lysis buffer (Hepes 10 mM, MgCl_2_ 1.5 mM, KCl 10 mM, DTT 0.5 mM, NP-40 0.05% with protease inhibitors (Roche)), followed by sonication and centrifugation. Protein concentration in the supernatant was quantified as explained above. Briefly, 100 µg proteins were incubated with reaction buffer containing 0.1 mM NADPH (Sigma) and 1 mM MG. NADPH reduction was followed spectrophotometrically at 320 nm for 1 h. NADPH quantity processed was calculated based on a calibration curve. Results were expressed as mmoles of NADPH transformed per hour by mg of protein extract.

### Statistical analysis

All experiments were performed as several independent biological replicates. All results were reported as means with standard error of the mean (SEM) as indicated in figure legends. Two group comparisons were performed using unpaired student’s t-test with or without Welsch’s correction according to homoscedasticity. When an experiment required comparisons between more than two groups, statistical analysis was performed using one-way or two-way ANOVA depending on the number of grouping factors. Dunnet’s or Bonferroni’s test were applied for simple or multiple comparisons, respectively. Outliers were detected using whisker box plots. In all cases, a bilateral p < 0.05 was considered as statistically significant with a 95% confidence interval.

## Electronic supplementary material

Supplementary figures
