# Supplementary material for: Hormetic potential of methylglyoxal, a side-product of glycolysis, in switching tumours from growth to death
Source: Sci Rep. 2017 Sep 15;7:11722. doi: 10.1038/s41598-017-12119-7 (PMC5600983; doi:10.1038/s41598-017-12119-7)
Supplement: Supplementary file 1 — Supplementary figures [file 41598_2017_12119_MOESM1_ESM.docx]

**SUPPLEMENTARY MATERIAL:**

**Supplementary Figures 1 to 6 and Table 1**

**Hormetic potential of methylglyoxal, a side-product of glycolysis, in switching tumours from growth to death**

Marie-Julie Nokin^1#^, Florence Durieux^1#^, Justine Bellier^1^, Olivier Peulen^1^, Koji Uchida^2^, David A. Spiegel^3^, James R. Cochrane^4^, Craig A. Hutton^4^, Vincent Castronovo^1^ and Akeila Bellahcène^1^*

^#^ These authors contributed equally to this work.

* Corresponding author

^1^Metastasis Research Laboratory, GIGA-CANCER, University of Liège, Liège, Belgium

^2^Laboratory of Food Chemistry, Department of Applied Biological Chemistry, Graduate School of Agricultural and Life Sciences, University of Tokyo, Tokyo, Japan

^3^Department of Chemistry, Yale University, 225 Prospect Street, New Haven, Connecticut, USA

^4^School of Chemistry and Bio21 Molecular Science and Biotechnology Institute, University of Melbourne, Australia

***Corresponding author:**

Akeila BELLAHCENE

University of Liège, GIGA-CANCER

Metastasis Research Laboratory

Pathology Tour, +4 level, Building 23

4000 Liège, BELGIUM

Tel. +32 4 366 25 57

Fax +32 4 366 29 75

a.bellahcene@ulg.ac.be

**LEGENDS TO SUPPLEMENTARY FIGURES**

**Supplementary Figure 1 (related to Figure 3).** ROS accumulation upon MG treatment was assessed by flow cytometry using CM-DCFDA probe in the indicated cancer cells. Data are presented as mean values ± SEM of three independent experiments. ns = not significant.

**Supplementary Figure 2 (related to Figure 3). GLO1 expression and activity in glioblastoma and breast cancer cell lines. (A).** GLO1 mRNA level was evaluated by RT-qPCR in U87-MG, U251, MDA-MB-231 and MCF7 cells cultured in low (LG) and high (HG) glucose medium. **(B).** GLO1 and Nrf2 protein levels were estimated using immunoblotting, with β-actin as a loading control. Immunoblots are representative of three independent experiments. **(C).** GLO1 maximal activity in the indicated cell lines. Data are presented as mean values ± SEM of three independent experiments. ns = not significant. Full-length blots are presented in Supplementary Figure 6.

**Supplementary Figure 3 (related to Figure 5). Cancer cells display a biphasic dose response growth curve upon MG treatment*.*** MDA-MB-231 cells were grown on the chicken chorioallantoic membrane (CAM) and treated daily with the indicated doses of MG. After 7 days, tumour volumes were calculated. Data are presented as mean values ± SEM of at least 10 eggs for each experimental condition. *p<0.05 and **p<0.01.

**Supplementary Figure 4 (related to Figure 2C).** Full-length blots.

**Supplementary Figure 5 (related to Figure 3C).** Full-length blots.

**Supplementary Figure 6 (related to Supplementary Figure 1B).** Full-length blots.

**Nokin, Durieux et al., Supplementary figure 1
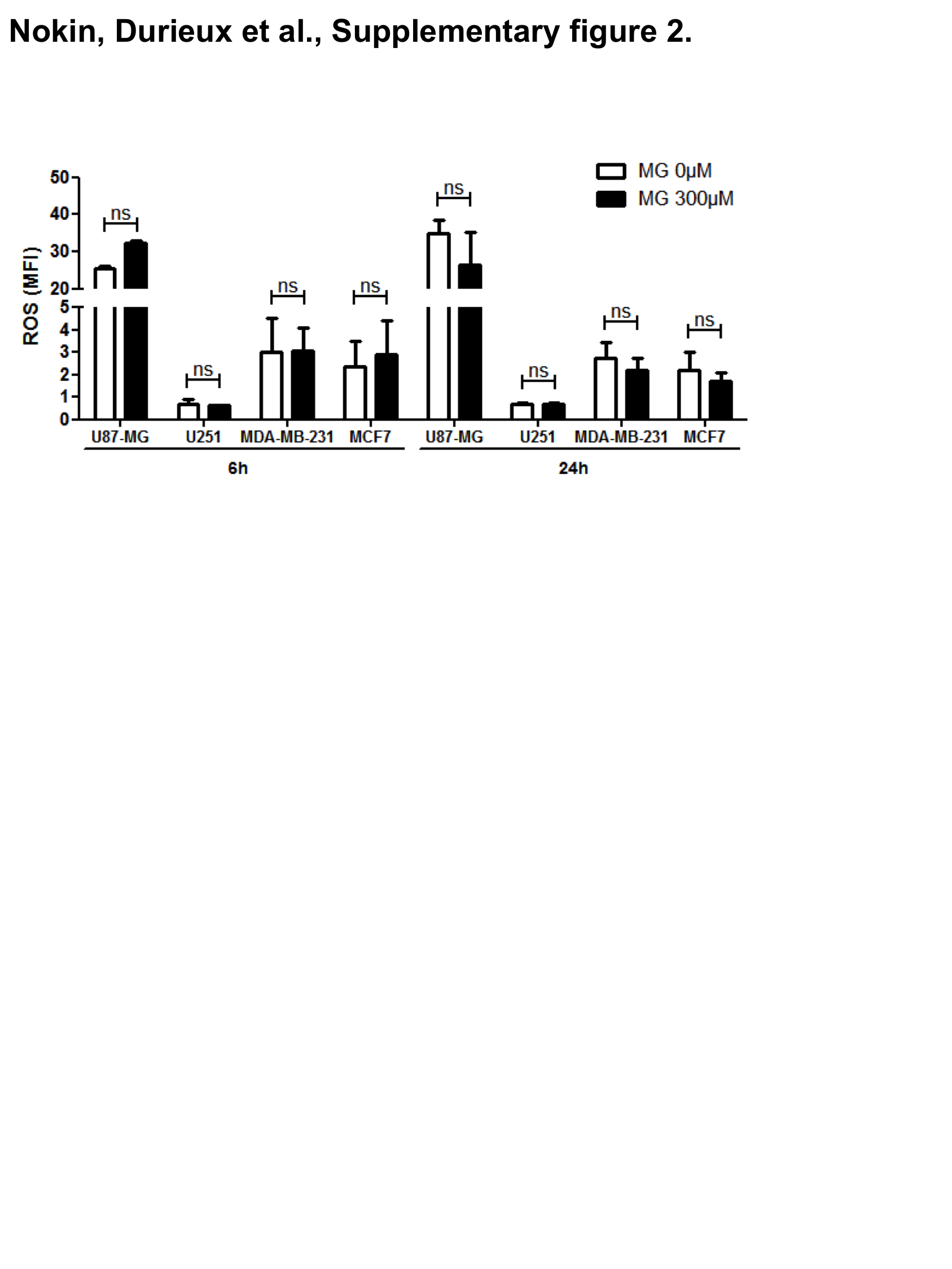
**

**Nokin, Durieux et al., Supplementary figure 2
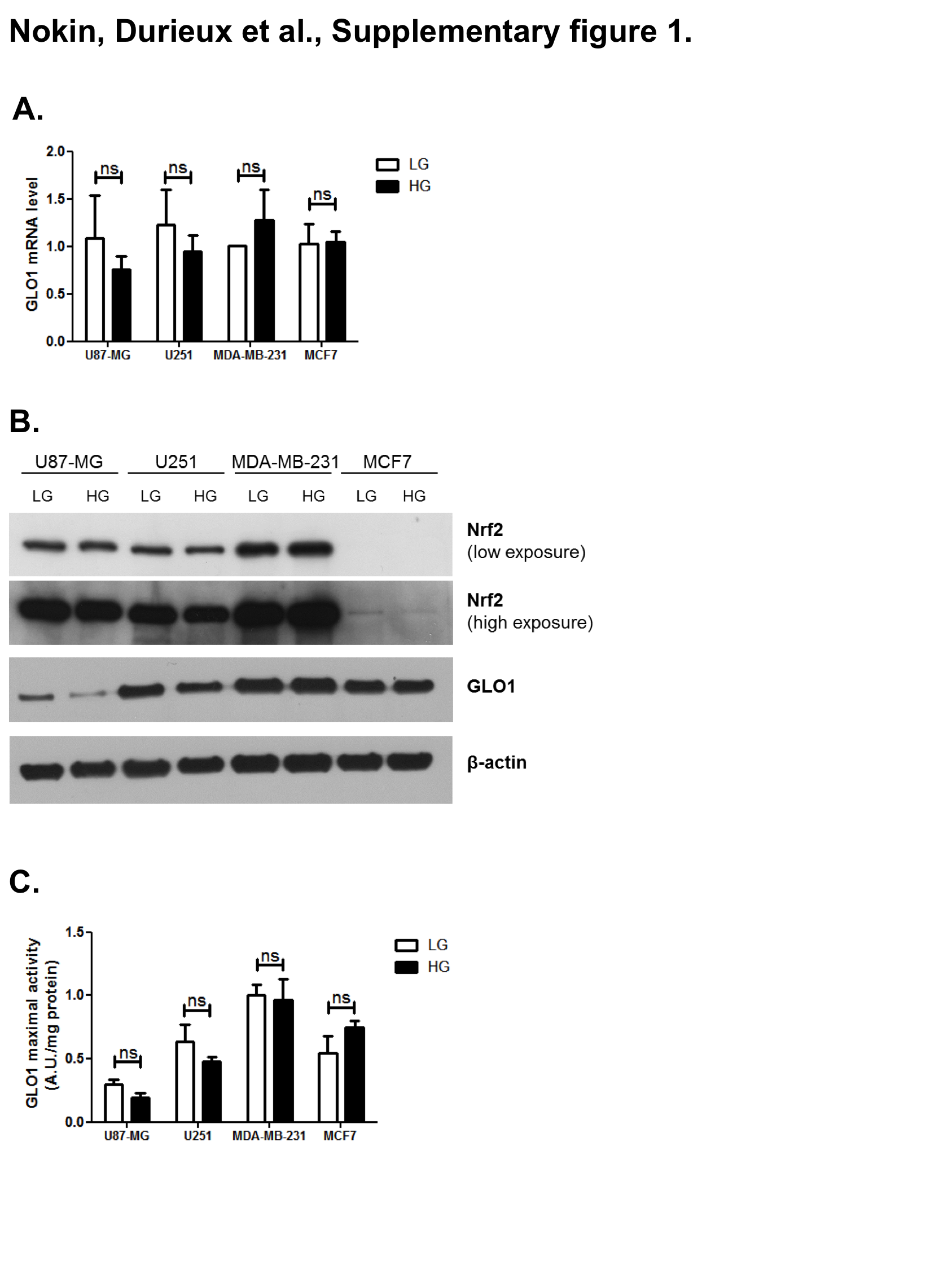
**

**
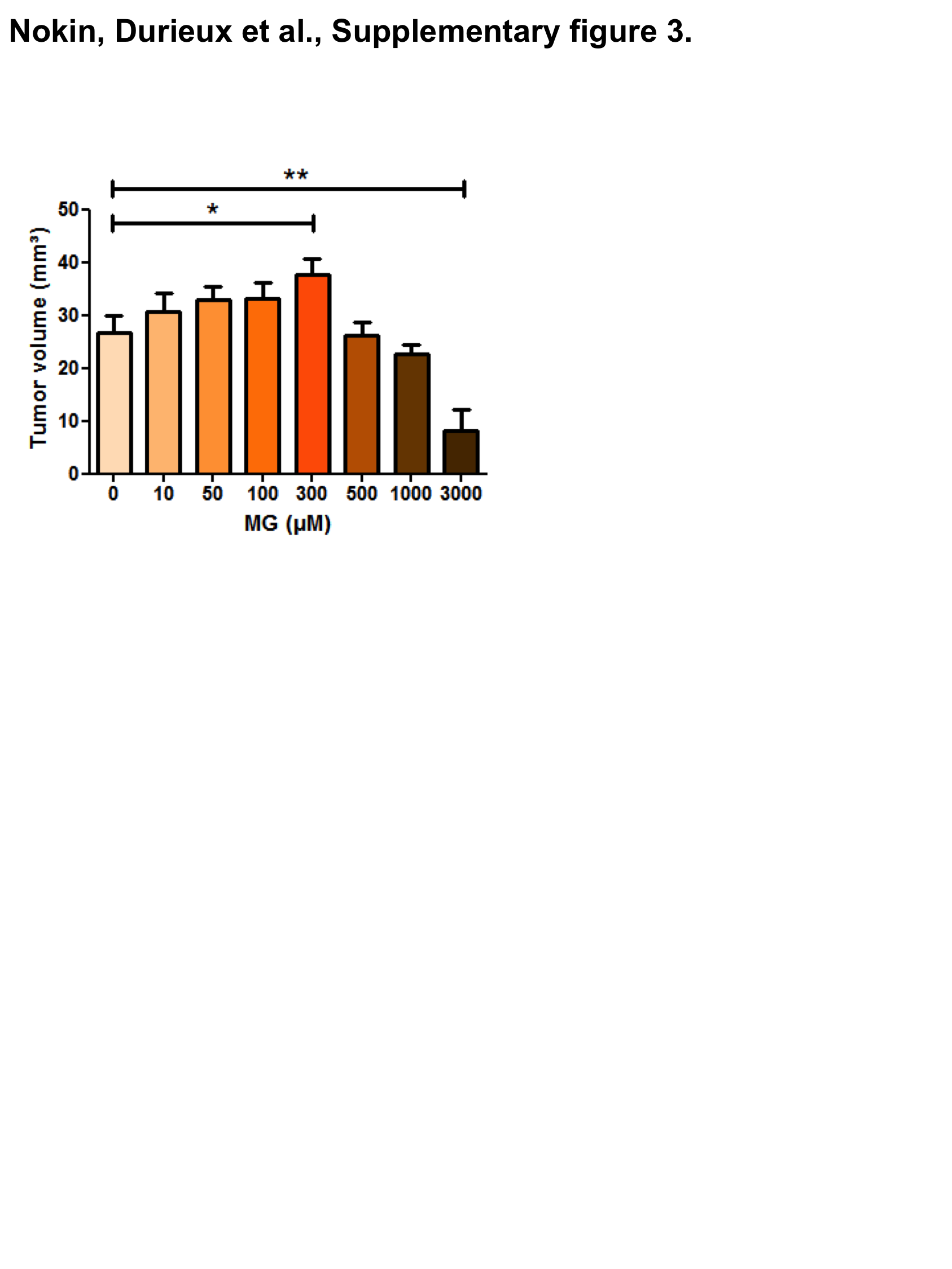
**

**
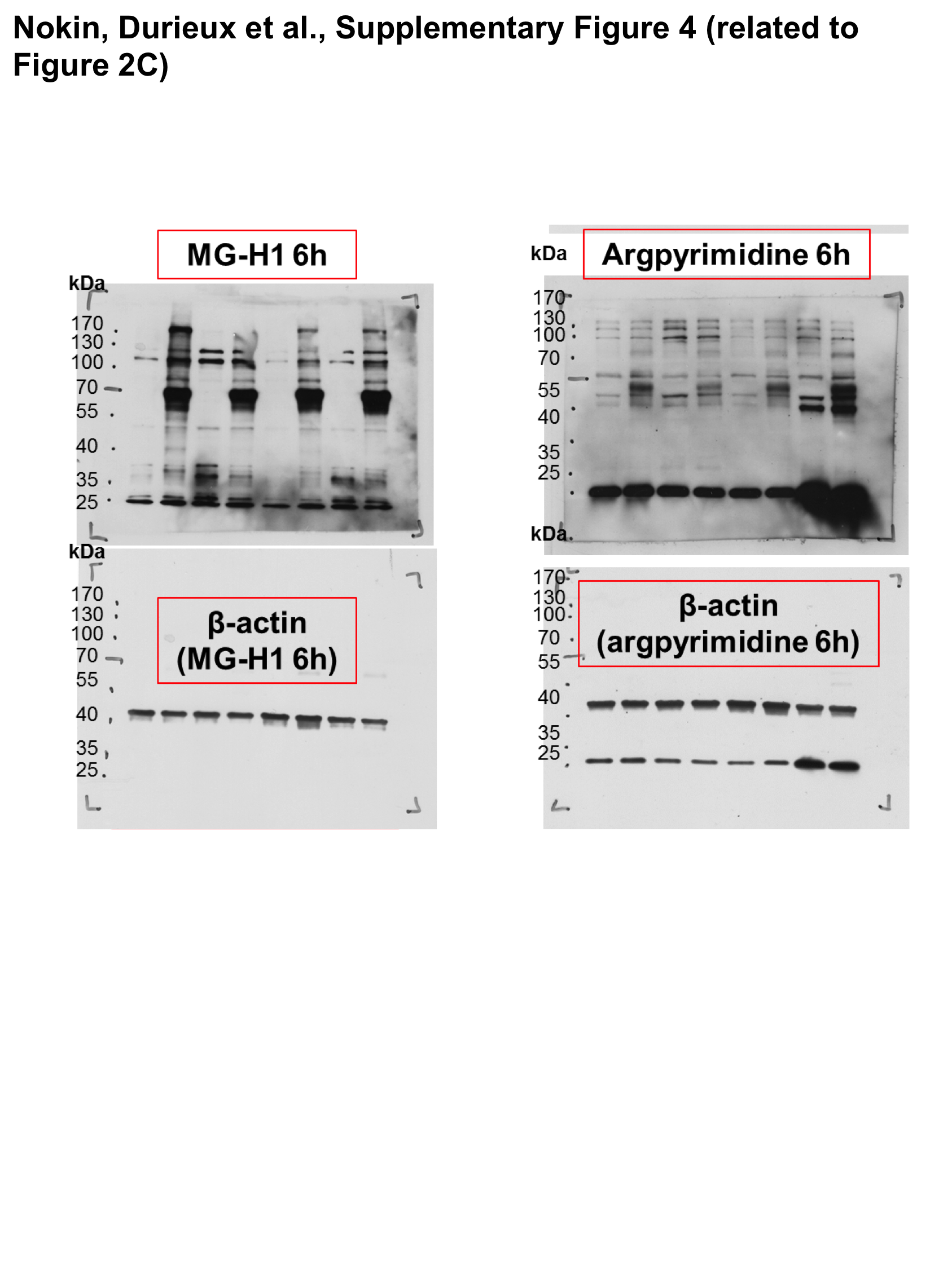
**

**
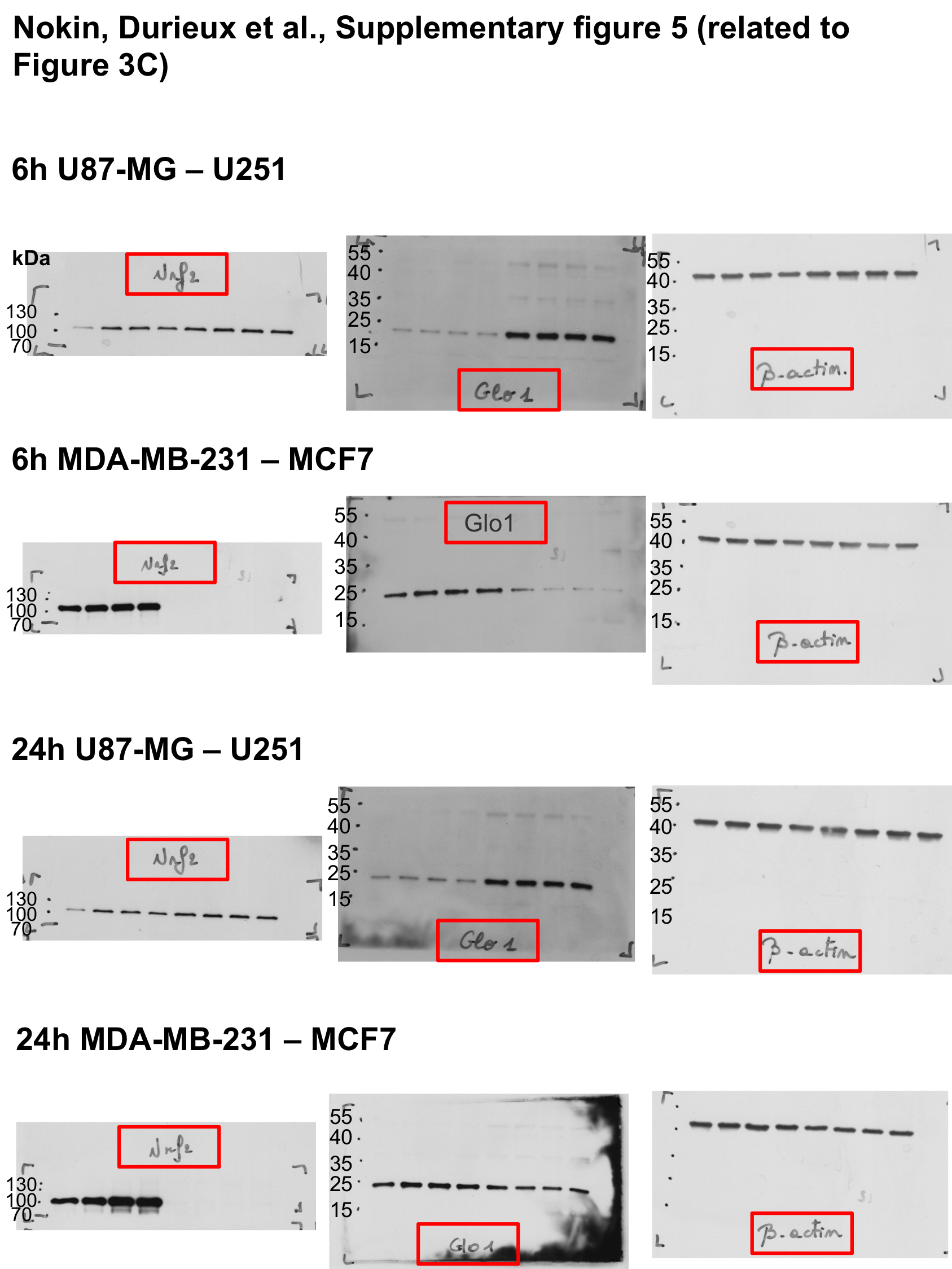
**

**
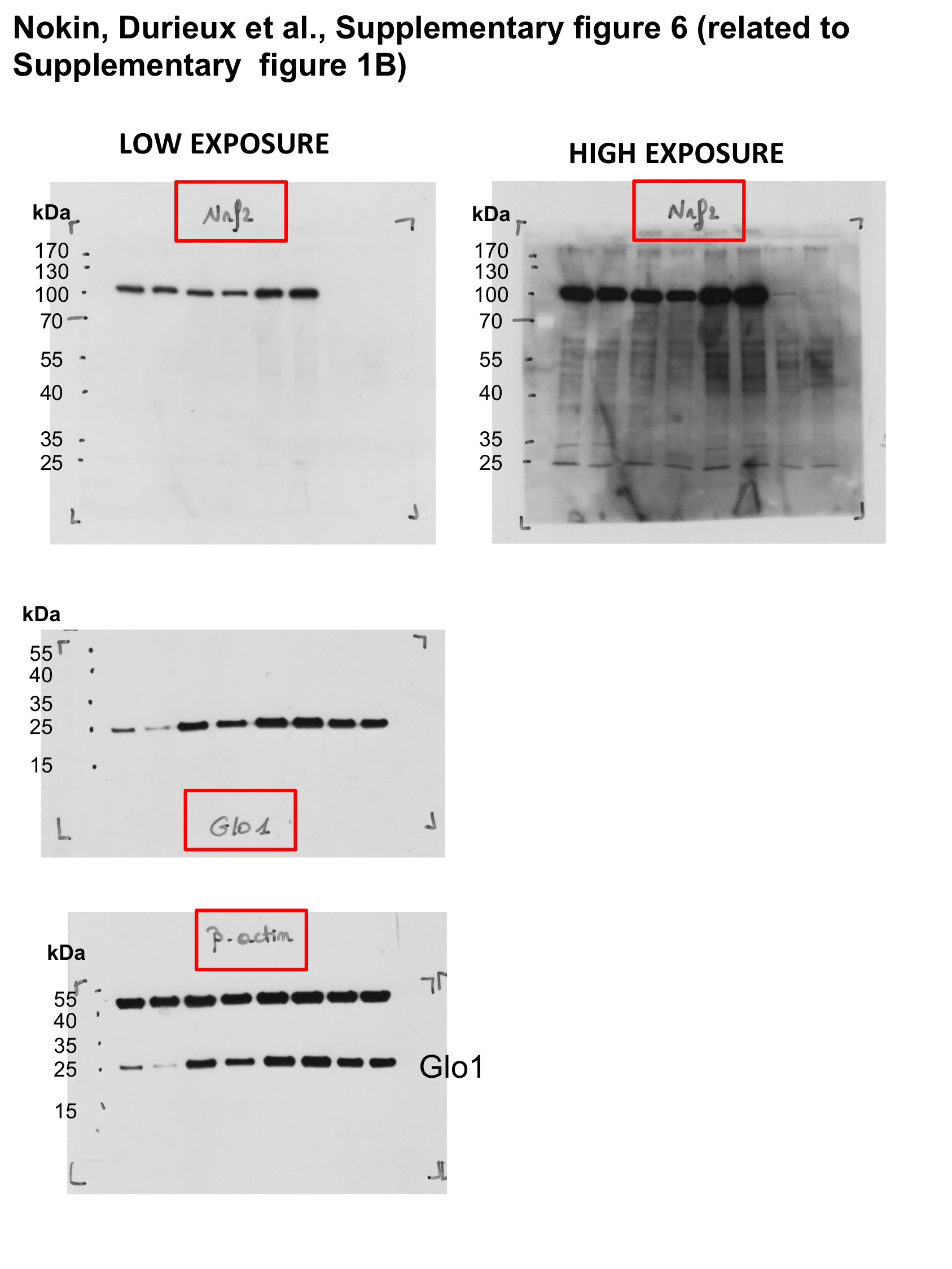
**

**Supplementary Table 1.** RT-qPCR primers sequences.

| **Name** | **Fw/Rv** | **Sequence** | **Probe (UPL, Roche)** |
| --- | --- | --- | --- |
| **AKR1B10** | Fw | 5’- AAAGCAACGTTCTTGGATGC -3’ | 17 |
|  | Rv | 5’- TGGAAGTGGCTGAAATTGG -3’ |  |
| **AKR1C1** | Fw | 5’-CATGCCTGTCCTGGGATTT -3’ | 49 |
|  | Rv | 5’- AGAATCAATATGGCGGAAGC-3’ |  |
| **AKR1C3** | Fw | 5’-CATTGGGGTGTCAAACTTCA -3’ | 27 |
|  | Rv | 5’- CCGGTTGAAATACGGATGAC-3’ |  |
| **18S** | Fw | 5’-CTTCCACAGGAGGCCTACAC-3’ | 46 |
|  | Rv | 5’-CGCAAAATATGCTGGAACTTT-3’ |  |
| **GLO1** | Fw | 5’-TGGCTTATGAGGATAAAAATGACA-3’ | **SYBR Green** |
|  | Rv | 5’-CAGCTCAAGTGTAGCTTTTCTGG-3’ |  |
| **Nrf2** | Fw | 5’-GAGAGCCCAGTCTTCATTGC -3’ |  |
|  | Rv | 5’- TCGTCAATGTCCTGTTGCAT-3’ |  |
